# Rhazes' Prescriptions in Treatment of Gout

**Published:** 2012-02-01

**Authors:** S Changizi Ashtiyani, A Golestanpour, M Shamsi, S M Tabatabaei, M Ramazani

**Affiliations:** 1 Department of Physiology, Arak University of Medical Sciences, Arak, Iran; 2 Department of Internal Medicine, Arak University of Medical Sciences, Arak, Iran; 3 Department of Public Health, Arak University of Medical Sciences, Arak, Iran; 4 Medical Ethics and History of Medicine Research Center, Tehran University of Medical Sciences, Tehran, Iran; 5 Department of Internal Medicine, Baqiatollal University of Medical Sciences, Tehran, Iran

**Keywords:** Gout, Medicine, History, Rhazes

## Abstract

**Background:**

Among the diseases that clinicians deal with, few do have a documented medical history that can be traced back to several centuries ago. A careful study of Rhazes’ Treatments on Gout reveals a lot about the nature and therapy of gout.

**Methods:**

We managed to study the perceptions about pathogenesis, symptomatology, diagnosis, and treatment of gout that have changed over time. We also discussed some of the past and present fallacies regarding this disease.

**Results:**

Rhazes provided a detailed description on the vital role of genetics and the relationship between the development of gout, an indulgent way of living, and tophi at a period of time between 1st and 6th centuries AD.

**Conclusion:**

This study showed that the findings of Rhazes about treatments of gout were consonant with modern medical theories.

## Introduction

Gout is a metabolic disease, which is characterized by acute or chronic arthritis and deposition of monosodium urate crystals in joints, bones, soft tissues, and kidneys.[1] Historically known as "the disease of kings" or "rich man's disease",[2] gout is an inflammatory arthritis that has been recognized since ancient times. The first written description of gout dates back to 2600 BC, when Egyptians noted the gouty arthritis of the big toe. Around 400 BC, the Greek physician, Hippocrates, also commented on gout.[3] Writing ca. 30 AD, Aulus Cornelius Celsus happened to recognize many of the features of gout, including its relationship with a urinary solute, late onset in women, linkage with alcohol, and perhaps even prevention by dairy products: “Again thick urine, the sediment from which is white, indicates that pain and disease are to be apprehended in the region of joints or viscera. … Joint troubles in the hands and feet are very frequent and persistent, such as what occurs in cases of podagra and cheiragra. These seldom attack eunuchs or boys before coition with a woman, or women except those in whom the menses have become suppressed”.[4]

All patients with gout have hyperuricemia at some point. Thus, the diagnosis of gout is centered around the fundamental pathophysiologic events defining the clinical state: tissue deposition of urate crystals; and the accompanying inflammatory and degenerative consequences.[5][6]

Abu Bakr Mohammad Ibn Zakariya Razi, known in the west as Rhazes (865-925 AD), was born in the ancient city of Rayy, near Tehran, Iran. He was a renowned physician in medical history and not only did follow Hippocrates and Galen, but also greatly extended the analytical approach of his predecessors.[7] He was particularly known for his scientific methodology, having founded medicine on clinical examination rather than theoretical studies. It is commonly held that the development of medicine was contingent on the progress made in clinical, not theoretical research. Hence, Rhazes’ Al-Hawi, known as Liber Continens, in which he had recorded thousands of clinical cases and recommended therapies, was acclaimed as the most important encyclopedia of clinical medicine in world; this, in turn, entitled the book to gain such significance in the history of medicine.[8] However, it was in medical contributions including Al-Mansuriy, Al-Shukuk ala Jalinus; Kittab al-Quling, etc, in addition to Maqale fi al-Naqras (Treatise on Gout).[9] Rhazes comprehensive view is one of the outcomes of philosophy, a view that modern medical research lack.

## Materials and Methods

This study is a comparison of modern medicine with some chapters of Rhazes’s Treaties on Gout (Maqale fi al-Naqras). We used this book in its original language (Arabic)[10] along with its Persian and English translations. A comparison was made between the English translation of the book based on the Arabic version and its Persian translation to provide a more accurate text.

Rhazes stated all aspects and causes of gout, its differentiation, clinical features, methods of treatments and some dietary suggestions for treatment of this disease. In our study of the reason and causality of gout, we started with headings of short questions and set to answer them through the provision of brief and eloquent sentences from the reviews of Rhazes’ prescriptions for treatment of gout.

### What Are the Causes of Gout?

So far, several studies have shown that the most important risk factor for gout is a high level of serum uric acid which results in urate crystal formation in the articular, periarticular, and subcutaneous tissues. Furthermore, it includes some of the dietary risk factors such as meat, seafood, beer, wine, liquor, and purinerich vegetables.[11][12] Rhazes stated that “Gout is abnormal phlegm that through blood reaches the joints and involves them and they gradually get hardened and become stone like.[13] Furthermore, “Gout occurs as a result, namely, of the coexistence of two predisposing factors, namely stoutness of the body and good health and equal vigor of all its organs. Furthermore, gout occurs as a result of the aggregation of metabolic excess in the blood”.[10][14] Statements of Rhazes are consonant with the above mentioned studies.

Both in Al-Hawi (Rhazes) and Cannon Fi-Tibb (Avicenna), classifications of gout with joint diseases and sciatica and other pains of the joint are made in one chapter.

### What Are the Methods of Gout Treatment?

Today, several studies indicate that life style modifications such as reducing alcohol intake, losing weight gradually, and limiting protein and purine content in diet (which increase uric acid levels in blood), drinking plenty of water throughout the day, always keeping hands and feet well covered and warm in cold months (gout attacks are very common during the cold months) can go some way towards reducing the frequency or likelihood of gout incidence.[15][16]

Rhazes stated that management of gout can be achieved if these ten procedures are followed: i) Abstinence from restricted diet; ii) Compliance with fluid and dietary regimens regarding the emphasis on certain food types and drinks; iii) Administration of laxatives; iv) Stimulations of emesis; v) Bloodletting; vi) Application of water to the feet; vii) Treatment with salves and poultices; viii) Steam baths; ix) Taking preventive measures to avoid recurrence of gouty attacks; x) Prompt management of incipient gout using counter acting drugs and analgesics.[10]

Roddy et al. indicated that the prevalence and incidence of gout have increased in the last few decades because of life-style and dietary factors.[17] Here again, this idea is in agreement with that of Rhazes’

### What Are the Dietary Restrictions for Gout Suffer?

Several studies have shown that people suffering from gout should avoid the following foods and beverages: Anchovies, bacon, goose, heart, liver, mussels, mutton, pheasant, sardine, sweet breads, salmon, scallops, trout, turkey, veal, and yeast. In fact, it is best to restrict all kinds of alcohol, because it can increase the level of uric acid in the body.[18][19]

Rhazes stated that “Gouty patients should forsake camels meat, beef, namaksud (salted jerked meat), as well as died game meat and all kinds of jerked meat. As regards fish, it is advisable to avoid all kinds of salted fish, as well as stinking rigid-flesh unsalted fish. Dairy products should be all forsaken except for small amounts of milk, cooked with rice and sprinkled with pinch of tabarzad (solid white sugar) sugar

It is also advisable to refrain from increased intake of certain types of dried fruits viz. walnuts, dried dates, unripe dates, honey Natif, as well as all kinds of Natif, pine seeds, Syrian carob beans, etc.[10]

### What Are the Dietary Recommendations for Gout Suffers?

Several studies suggest the following dietary recommendations for gout:

Fresh cherries, strawberries, blueberries, and other red-blue berries, bananas, celery, tomatoes, vegetables, including kale, cabbage, parsley, green-leafy vegetables, foods high in bromelain (pineapple) ?foods rich in vitamin C, fruit juices and purified water, low-fat dairy products, coffee, tea, carbonated beverages, and essential fatty acids (tuna, salmon, flaxseed, nuts, and seeds).[18][20][21]

Rhazes stated that “Cereals are generally not recommendable; however, the least pernicious of which are beans and gram peas for gout sufferers having biliary blood, and rice and chickpeas for patients with phlegmatic blood. Furthermore, eggs are restricted unless they are soft-boiled and eaten by sipping. As for dried fruits, the most recommendable are almonds. Patients with gout are allowed to have fresh fruits of moderate sweetness, such as fully ripened grapes, figs, apples, pomegranate, quince and pears. Vegetables are totally restricted except for lettuce, endives, dodder and celery for they have least harm to gouty patients”.[10]

Rhazes carefully elaborates on the role of temperature and duration of hydrotherapy in treatment of gout and reduction of pain associated with it, and sets to include and compare them with the ideas of other scientists of his era.

### How Can Gout be treated By the Application of Water to the Feet?

Drinking plenty of fluids reduces the uric acid from body and thus, prevents joint inflammation. It is not enough if one drinks water whenever one feels thirsty. Patients must drink water all over the day even if they are not thirsty. Along with drinking eight and ten glasses of water on a daily basis, patients should also eat fruits that are rich in water content like watermelon. Eating watermelon and its seeds will also help expel the uric acid from body.[22][23][24]

Rhazes stated that “Two factor are involved in gout management via application of water to the feet; the temperature of the water and the time of application. Some patients with gout are advised to use extremely cold water during acute episodes, while others need to apply tepid or hot water. Patients with gout precipitating from hot pungent humors should pour cold water on their feet at the onset of the illness; meanwhile, cold water strengthens the organs and helps them reject the wastes that jab their way into them. Tepid water, on other hand, expels waste deposits from the affected organ however; the resulting heat may draw another kind of waste matter to it. Gouty patients, who have stout bodies harboring a surplus of wastes, should refrain from using hot water at an early stage. At the onset of pain, it is favorable to apply cold rather than hot water to the feet. Hippocrates has prescribed a treatment for gout by the application of cold waters to the feet but the never mentioned the application of hot water as a possible remedy.[10]

Rhazes' application of a combination of remedies, potions, and drugs which was due to his clinical experiences calls for a more elaborate consideration. In fact, so many of treatment and pain relief mechanisms of these drugs are still in need of further research with regard to their contents and combinations.

### How to Stop Progression of Incipient Gout Using Counteracting Drugs and Analgesics?

Incipient gout should be treated using purgatives at the onset of an attack; porcelain water has the strongest effect for patients with gout resulting from biliary blood, whereas gout resulting from viscid phlegmatic blood may be controlled using purgative jawarisnat such as tuffahiyyat, kummitihraiy, safarjaliy, tamiry, etc.[10]

In addition to presenting various recommendations for treatment of gout and controlling its progress, Rhazes effectively discussed the methods for prevention of the recurrence of gout, which indeed indicated the depth of his knowledge about this disease and its development.

### How to Prevent Gout Recurrence?

Once the attack has passed, the next step is to help prevent recurrences by addressing triggering factors that can be modified. Patients are best advised to lose weight gradually and progressively (if they are obese), to reduce alcohol consumption and intake of sugar-sweetened soft drinks, and to eat smaller amount of purine-rich foods.[25] Such life style modifications can work if these environmental risk factors are present and prescription of regular urate-lowering medication is not always necessary. However, if the blood uric acid level remains high and if acute attacks continue then long term joint damage is very likely.[26]

Rhazes stated that “Prevention of gout can be achieved by abstinence from unhealthy foods and cutting down on healthy food types as prescribed before; the other means is expulsion of humoral excess that might aggregate in the body, especially after a healthy meal or excessive intake of food”.[10]

## Discussion

Gout was described as the "King of diseases and disease of Kings". The highest incidence of gout occurs between 30 and 45 years of age in men, and between 55 and 70 in women. Thus, the clinical manifestations of hyperuricemia occur, on average, about two decades later than the initial physiologic increase in serum urate concentration. This observation suggests that there is a lengthy period of asymptomatic hyperuricemia preceding the occurrence of gout in both men and women.[27] Gout can be induced due to excessive production of and/or inadequate excretion of uric acid. Of all the uric acid excreted daily, one third is yielded through the diet, and the rest is generated by the resources residing in the body.[28] Nearly, ten percent of gout cases are due to the excessive production of uric acid while 90% of cases are induced by decreased excretion of uric acid. In his description of the causes of the disease, Rhazes highlights the deposition of waste substances in joints.[14]

Nowadays, owing to the application of modern technology in medicine, the deposition of crystals of uric acid has come to be known as the main factor in Pathophysiology of patients.[29] A purine limited diet, which is prescribed as a contributing factor in treatment of gout and other abnormalities that affect purine metabolism, is targeted to reduce uric acid level in body. In such a diet, the consumption of purine-rich sources of nutrition such as liver, glandular organs, fish and other types of seafood, meat, beans, and spinach are attenuated according to the conditions of the patient, and the consumption of these foods will not be allowed during acute attacks of gout.[30] In recent years, due to the availability of such effective drugs as allopurinol which can inhibit the production uric acid, these diets have received little attention. In fact, using diets which contain low amounts of purine can only reduce serum uric acid concentration up to 1 mg/dl, which is not significant in comparison to present drugs.

Since controlling and maintaining a proper bodyweight is of particular importance for such patients, they should try to keep their weight at an ideal level through observing a balanced diet and performing physical exercises, especially walking. For diluting the concentration of urine, accelerating its excretion, preventing urea deposition in kidneys and thus formation of kidney stones, drinking large amounts of water and other liquids is recommended.[31] In the his composition of medical texts, in addition to provision of descriptions about the causes, manifestations, and the methods of treatment of diseases, Rhazes tried to gather and quote the ideas and arguments of all ancient and modern time physicians, from Hippocrates and Galen to Issac Ibn Honein, about each disease.[14]

Regarding gout, Rhazes comes to propose some guidelines for its treatment. First, he suggested keeping a certain diet for decreasing the amount of waste material produced in the body. It seems that Rhazes aimed in prescription of such procedures as phlebotomy and enema was to decrease the waste material concentration; however, these methods are of little application due to the existence of better method of treatment.[14] The next solution that Rhazes offered was abstinence from situations that could lead to the acute attack of the disease.[14]

This subject, it appears, is still of significance in modern medicine; however, there are a lot of unanswered questions in this realm. For instance, the reasons why in a group of individuals with the same level of serum uric acid, acute attacks of gout happen only to some, and why under the same conditions, an individual does not always experience an attack, are still unknown.

In short, Rhazes tried to present methods of treatment for attenuating inflammation. He views soregan as one of the useful drugs which helps expel cold type of sputum. In other words, this drug absorbs the waste materials and prevents their return. Rhazes found this effect exclusive to soregan and believed that other warm drugs were not of such an effect. Nowadays, soregan is known as colchicum speciosum which contains colchicine which is still in the list of the most effective drugs in treatment of gout.[32]

Although through using such drugs as colchicine, glucocorticoids and non-steroid anti-inflammatory combinations, most of the patients get cured in a short period of time, it must be mentioned that despite the plethora of explanations about this matter, the main causes for cessation of joint inflammation with or without using these drugs are not completely known.[33]
